# Mortality risk of bloodstream infection caused by either *Escherichia coli* o*r Klebsiella pneumoniae* producing extended-spectrum β-lactamase: a prospective cohort study

**DOI:** 10.1186/s13104-019-4751-9

**Published:** 2019-11-01

**Authors:** Osman Sianipar, Widya Asmara, Iwan Dwiprahasto, Budi Mulyono

**Affiliations:** 1 Department of Clinical Pathology and Laboratory Medicine, Faculty of Medicine, Public Health and Nursing, Universitas Gadjah Mada, Radioputro Building 5th Floor, Jalan Farmako, Sekip Utara, Yogyakarta, Indonesia; 2 Department of Microbiology, Faculty of Veterinary Medicine, Universitas Gadjah Mada, Yogyakarta, Indonesia; 3 Department of Pharmacology, Faculty of Medicine, Public Health and Nursing, Universitas Gadjah Mada, Radioputro Building 2nd Floor, Jalan Farmako, Sekip Utara, Yogyakarta, Indonesia

**Keywords:** Bloodstream infection, *K. pneumoniae*, *E. coli*, Extended-spectrum β-lactamase, Mortality

## Abstract

**Objective:**

Several studies reported that infection of extended-spectrum β lactamase (ESBL)-producing *Escherichia coli* (*E. coli*) or *Klebsiella pneumoniae* (*K. pneumoniae*) contributed to higher mortality rates but others found it was not associated with mortality. A prospective cohort study which involved 72 patients was conducted to assess the risk of mortality of bloodstream infection due to ESBL-producing *K. pneumoniae* or *E. coli* as compared to those infected by either *K. pneumoniae* or *E. coli* which not produce ESBL.

**Result:**

Mortality in the group of patients infected with ESBL-producing bacteria was 30.6%, whereas in another group which was infected with non ESBL-producing bacteria was 22.2% (*p *= 0.59). Kaplan–Meier’s analysis showed that the survival rate during 14-days follow-up among these two group was not significantly different (*p* = 0.45) with hazard ratio 1.41 (95% CI  0.568–3.51). Stratification analysis found that adult and elderly patients, patients with sign of leukocytosis, and patients treated with carbapenem were modifier effect variables.

## Introduction

Bloodstream infection is known as the presence of positive blood culture with clinical signs and symptoms of infection for which contamination can be excluded. Gram negative rod bacteria are frequently found as the cause of infection [1, 2]. Among this group, *Escherichia coli* (*E. coli*) and *Klebsiella pneumoniae* (*K. pneumoniae*) are the most common bacteria reported as the etiology of bloodstream infection [1–4].

Antimicrobia resistance rates increased worldwide as reported in a recent World Health Organization (WHO) global report on antimicrobial resistance surveillance. *K. pneumoniae* and *E. coli* were 2 out of 7 species of bacteria in which antimicrobial resistance was surveyed [5]. These 2 clinical isolates could produce extended-spectrum β lactamase (ESBL) which could give rise to multi resistant. Recent study reported that the prevalence of ESBL-producing *K. pneumoniae* and ESBL-producing *E. coli* in Pekanbaru Indonesia were 66.2% and 62.2% respectively [6]. Another study conducted in Banda Aceh, Indonesia reported that prevalence of ESBL-producing *K. pneumoniae* and ESBL-producing *E. coli* were 80% and 85% respectively [7].

Risk of mortality was reported higher among patients who were infected with resistant strains of microbes [5, 8, 9]. Mortality rates of patients infected by ESBL-producing *E. coli* or *K. pneumoniae* were higher compared to those infected by non ESBL-producing *E. coli* or *K. pneumoniae* [10, 11]. But some studies reported that risk of mortality of patients infected by ESBL-producing *E. coli*/or *K. pneumoniae* was not statistically different compared with those infected by non ESBL-producing *E. coli* or *K. pneumoniae* [12–15]. The aim of this study was to assess the risk of mortality of bloodstream infection due to ESBL-producing *K. pneumoniae* or *E. coli* as compared to those infected by either *K. pneumoniae* or *E. coli* which not produce ESBL.

## Main text

### Methods

This was a prospective cohort study that involved 72 patients who suffered from bloodstream infection caused by either *E. coli* or *K. pneumoniae* with Pitt bacteremia score less than 4. This score was measured according to previous study [10]. The subject of the study were recruited consecutively up to number of sample in each group were sufficient. One group consisted of 36 subjects infected by ESBL-producing *E. coli* or *K. pneumoniae* (exposed group), whereas another group consisted of 36 subjects infected by non ESBL- producing *E. coli* or *K. pneumoniae* (un-exposed group). These 2 groups were observed prospectively for 14 days starting right after diagnosis was determined based on positive blood culture to assess patient survived or died during this period. Mortality was defined as the suspension or cessation of vital processes of the body, as heart beat and respiration. Exposure was bloodstream infection caused by ESBL-producing *E. coli* or *K. pneumoniae*. It was determined whenever one set blood culture (2 bottles) gave consistent result of bacterial growth of either *E. coli* or *K. pneumoniae*. Identification of these isolates and also ESBL-producing bacteria was done by microdilution broth method using Vitek 2 system.

Blood samples were inoculated into 2 bottles aerobic blood culture media (ratio sample: media approximately 1:10), and then incubated in an automatic incubator. After the growth of bacteria was detected, samples were further processed for gram staining and microscopic examination as well as sub-cultured onto McConkey and blood agar media. Identification and antimicrobial susceptibility tests were conducted from the colony that grew on these 2 media. Identification of isolates were performed using the Vitek 2 system.

Source of laboratory data were from both Clinical laboratory of Dr. Sardjito and Panti Rapih Hospitals. Whereas source of clinical data was from both Medical Record Unit of Dr. Sardjito and Panti Rapih Hospitals. A clinical research form was used to collect data from each subject of study.

Sample size was calculated based on mortality of patients suffer from bloodstream infection caused by ESBL-non producing *E. coli* or *K. pneumoniae* 15%. Risk of death among those suffer from bloodstream infection caused by ESBL producing *E. coli* or *K. pneumoniae* 3 times. We considered power of the study 80%, confidence interval of 95%, and 10% drop out. Finally we defined number of subject in each group (expose and unexposed) was 36 subject.

Data collected were analyzed by descriptive statistics. Difference in mean and proportion among exposed and un-exposed group were tested using independent *t* test and Chi square test respectively. Survival analysis was conducted using Kaplan–Meier analysis in order to analyze probability of surviving in 14 days of both exposed and un-exposed group. Stratification analysis was conducted using Mantel Haenzel test to identify modifier effect variable.

### Results

#### Characteristics of study subjects

Subjects of this study consisted of 44 males and 28 females. The clinical diagnosis/condition of the study subjects as the reason for blood culture was mostly suspected sepsis. Among those infected by the ESBL producing bacteria 63.9% were diagnosed as sepsis, 13.9% each were diagnosed as SIRS and other infections, and 8.3% suffered from fever. Meanwhile among those infected by ESBL non-producing bacteria, 36.1% were diagnosed as sepsis and other infections, 19.4% diagnosed as systemic inflammatory response syndrom (SIRS), and 8.3% were diagnosed as fever.

The underlying diseases or comorbidities among study subjects were mostly due to malignant disease, and chronic illness, or malignant disease and chronic diseases. Among those infected with the ESBL producing bacteria, 27.8% suffered from malignant disease, 16.7% each suffered from chronic illness, and congenital disease, 8.3% each suffered from cholestasis, and malignant disease mixed with chronic diseases. Meanwhile the underlying disease/comorbidity among those infected by the ESBL non-producing bacteria were as follows: 33.3% suffered from malignant disease, 22.2% suffered from chronic disease, 13.9% suffered from malignant disease and chronic diseases, 8.3% suffered from chronic and endocrine diseases, and 5.6% suffered from cholestasis. The underlying disease/comorbidity which was categorized as other diseases accounted for 19.4% among those infected by the ESBL producing bacteria, and 11.1% among those infected by ESBL non-producing bacteria (Table 1).

**Table 1:** Comparison between ESBL and non-ESBL producing of *E. coli* or *K. pneumoniae* based on clinical variables

No	Variable	Bloodstream infection due to either *E. coli* or *K. pneumoniae*		*p*
		ESBL-producing	Non ESBL-producing	
1	Type of ward, n (%)			0.32
	General	9 (25.0)	12 (33.3)	
	Malignancy	7 (19.4)	12 (33.3)	
	Intensive	12 (33.3)	5 (13.9)	
	Surgery	3 (8.3)	3 (8.3)	
	Infection	2 (5.6)	3 (8.3)	
	Other	3 (8.3)	1 (2.8)	
2	Length of stay (day)			0.85
	Mean	10.52	11.21	
	SD	15.77	15.58	
3	Diagnosis, n (%)			0.08
	Sepsis	23 (63.9)	13 (36.1)	
	SIRS	5 (13.9)	7 (19.4)	
	Fever	3 (8.3)	3 (8.3)	
	Other infection	5 (13.9)	13 (36.1)	
4	Underlying disease/comorbid, n (%)			0.09
	Malignant disease	10 (27.8)	12 (33.3)	
	Chronic disease	6 (16.7)	8 (22.2)	
	Malignant and chronic diseases	3 (8.3)	5 (13.9)	
	Chronic and endocrine disease	0 (0)	3 (8.3)	
	Cholestasis	3 (8.3)	2 (5.6)	
	Congenital disease	6 (16.7)	0 (0)	
	Other	7 (19.4)	4 (11.1)	
5	Survival in 14 days, n (%)			0.59
	Alive	25 (69.4)	28 (77.8)	
	Died	11 (30.6)	8 (22.2)	
6	Source of infection, n (%)			0.60
	Unknown	14 (38.9)	20 (55.6)	
	Lung	8 (22.2)	4 (11.1)	
	Gastrointestinal tract	3 (8.3)	4 (11.1)	
	Urinary tract	4 (11.1)	3 (8.3)	
	Skin	3 (8.3)	1 (2.8)	
	Other	4 (11.1)	4 (11.1)	

*ESBL* extended-spectrum β lactamase, *SD* standard deviation, *SIRS* systemic inflammatory response syndrome

During 14 days follow-up, after bloodstream infection caused by either *E. coli*/*K. pneumoniae* was determined, 53 subjects remained alive, while 19 subjects died (26.4%). The mortality among those infected by the ESBL producing bacteria was 11 subjects (30.6%), while mortality among those infected by ESBL non-producing bacteria was 8 patients (22.2%). The proportion of death among these 2 group was not significantly different (*p * = 0.59).

The underlying diseases/comorbidities of 11 cases of death in patients with bloodstream infections due to *K. pneumoniae* was mostly malignant disease (5 subjects), followed by chronic illness (3 subjects), postoperative complication (1 subject), congenital disease (1 subject), and Human Immunodeficiency Virus (HIV) disease stage 3—malnutrition (1 subject). Similarly, the underlying diseases/co-morbidities of 8 cases of death in bloodstream infections due to *E. coli* were mostly malignant disease (5 subjects), followed by chronic diseases (3 subjects).

#### Survival analysis

Among those who suffered from bloodstream infection caused by the ESBL producing bacteria, mortality was 30.6% subjects (11/36). Whereas mortality among those infected by non ESBL-producing bacteria was 22.2% (8/36).

**Fig. 1:**
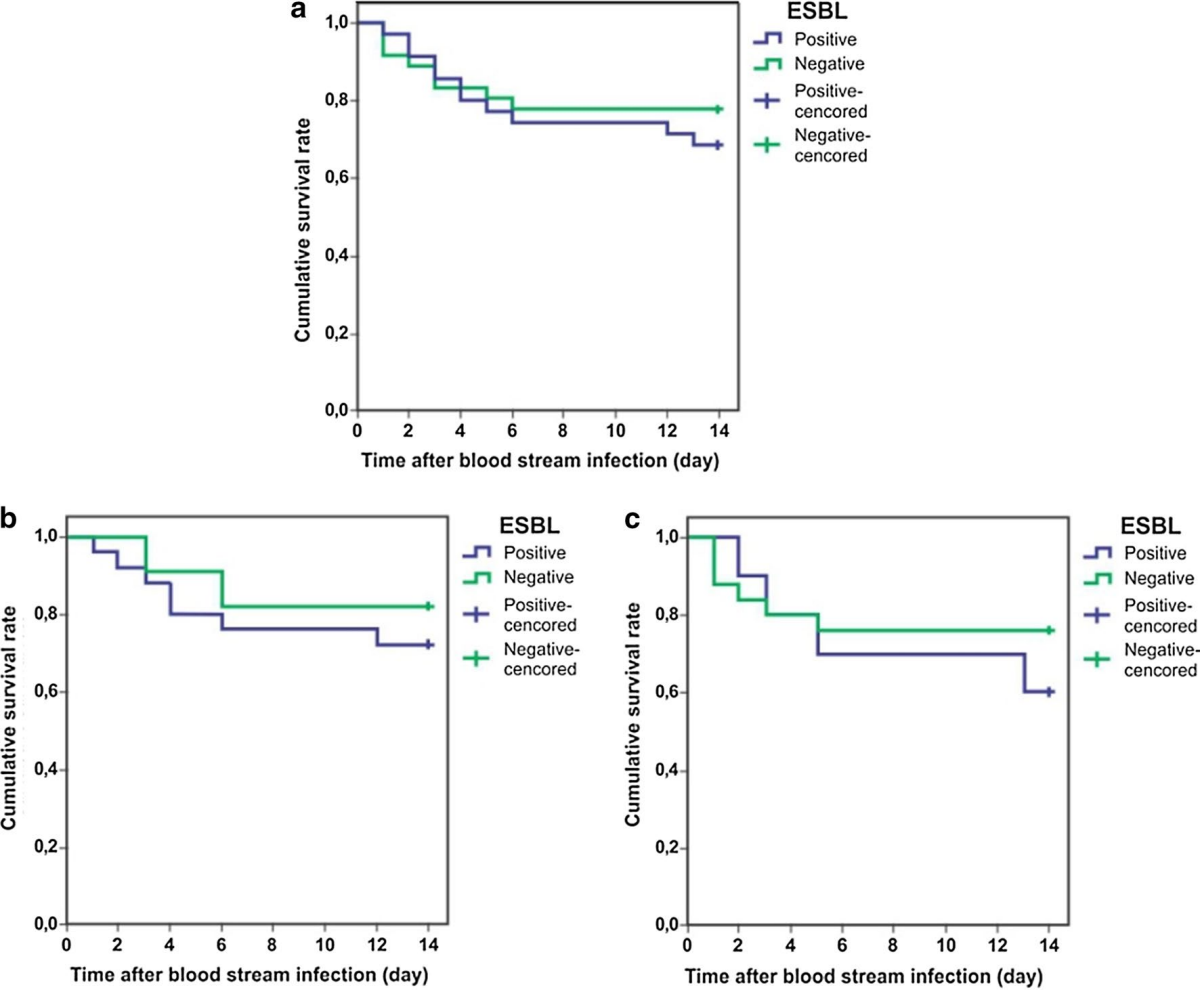
Survival analysis. **a** Survival of those infected by either *E. coli or K. pneumoniae;* **b** Survival of those infected by *K. pneumoniae*; **c** Survival of those infected by *E. coli*

Kaplan–Meier’s survival analysis results (Fig. 1) showed that the survival of these two groups did not differ significantly (*p* = 0.450) with a hazard ratio of 1.41 (95% confidence interval = 0.57–3.51). The results of stratified analysis in those infected by *K. pneumoniae* showed that the survival rate was not significantly different among the patients infected by ESBL producing bacteria, and by ESBL non-producing bacteria (*p* = 0.53). In addition, stratification analysis among those infected by *E. coli* showed a similar result (*p* = 0.45).

#### Stratification analysis

This analysis was done in order to identify modifier variables. Eventhough, overall risk of mortality is not different significantly between exposed and un-exposed group but 3 modifier variables could be identified namely those patient with age group adult to elderly, those patient with sign of leukocytosis, and those patient treated with carbapenem. These 3 modifier effect variables were identified if the different between adjusted relative risk (RR_MH_) and relative risk crude (RR_crude_) was at least 10% [16] (Table 2).

**Table 2:** Stratification analysis to evaluate mortality risk among those infected by either *E. coli* or *K. pneumoniae* producing ESBL

Strata	RR_Strat_	95% CI	*p*	RR_Crude_	RR_MH_
Males	1.89	0.59–6.01	0.43	1.38	1.40
Females	0.84	0.2–3.55	1		
Neonates up to 17 years old	0.78	0.2–2.98	1	1.38	1.64
Adult and elderly	2.57	1.04–6.36	0.04		
Primary bloodstream infection	1.17	0.33–4.10	0.57	1.38	1.43
Secondary bloodstream infection	1.65	0.61–4.47	0.53		
Length of stay > 2 days (before bloodstream infection established)	2.13	0.71–6.38	0.29	1.38	1.25
Length of stay ≤ than 2 days (before bloodstream infection established)	0.62	0.21–1.79	0.68		
Infected by *E. coli*	0.96	0.29–3.17	0.95	1.38	1.47
Infected by *K. pneumoniae*	2.05	0.62–6.76	0.37		
Leukocytosis	3.60	1.44–9.02	0.01	1.38	1.68
Non-leukocytosis	0.52	0.13–2.04	0.91		
Neutropenia	0.75	0.11–5.18	1.00	1.38	1.45
Non-neutropenia	1.69	0.66–4.34	0.41		
Underlying disease, malignancies	1.31	0.48–3.58	0.90	1.38	1.46
Underlying disease, non malignancies	1.65	0.48–5.74	0.48		
Inappropriate antimicrobial prescription in definitive therapy	9.53	0.60–152.02	0.70	1.38	1.42
Appropriate antimicrobial prescription in definitive therapy	0.74	0.28–1.93	0.77		
Definitive therapy using carbapenem	3.10	0.21–46.34	0.29	1.38	1.63
Definitive therapy using non-carbapenem	1.25	0.49–3.21	0.45		
Empiric therapy using cephalosporin	1.10	0.39–3.10	1.00	1.40	1.39
Empiric therapy using non-cephalosporin	2.08	0.51–8.52	0.27		

*RR*_*strat*_ relative risk from stratification analysis, *RR*_*crude*_ relative risk from total sample, *RR*_*MH*_ relative risk from Mantel Haenzel analysis

### Discussion

The underlying diseases/comorbidities of the study subjects were mostly malignant disease, and chronic illness, or malignant disease and chronic diseases. Almost all of the study subjects have an underlying disease/comorbidity. This finding is similar to previous studies [8, 17–19].

In this study, overall mortality among exposed group was 26.4% (19/72). Another similar study reported that 28 day-mortality rate for patients with blood-stream infection was 24.6% (47/191) [19]. Flokas et al. in 2017 reported that mortality among neonates who suffer from bloodstream infection due to the ESBL producing *Enterobacteriaceae* was 36% [20]. In patients suffering from bacteremic pneumonia caused by ESBL-producing *E. coli* or *K. pneumoniae,* 30 day-mortality was reported as 40.5% (45/111) [21].

Mortality of those who suffered from bloodstream infection due to *E. coli* was 30.8% (8/26). Another similar study reported that 7 days-mortality was 8.5% (128/1499) among those who were infected by *E. coli* [15]. Thirty days-mortality of this *E. coli* infection was reported as high as 28.5% (101/354) [11]. Number of subject infected by ESBL-producing *E. coli* was 10 patients, and 3 of them (30.0%) died during 14 days observation, whereas the mortality among those infected by ESBL non-producing *E. coli* was 31.25% (5/16). Thirty days-mortality among those infected by ESBL producing *E. coli* as reported in other study were as follows 26.4% (14/53) [18] and 21.6% [22]. Another study reported 3 months-mortality as high as 18% (34/232) for older adults and 5.7% (7/145) for younger adults [23].

Mortality of those who suffered from bloodstream infection caused by *K. pneumoniae* was 23.9% (11/46). This mortality was lower compared with another study with 46.2% (48/104) [12]. Twenty-eight days-mortality of bloodstream infection caused by *K. pneumoniae* was reported in 2 studies as high as 47.9% (91/190) [24], and 22.8%, respectively [25]. Seven days-mortality of this infection was 9.4% (33/352) [15]. In this study, mortality among those infected by ESBL producing *K. pneumoniae* and ESBL non-producing *K. pneumoniae* were 30.8% (8/26) and 15.0% (3/20), respectively. Another study reported mortality rates among those infected by ESBL-producing *K. pneumoniae* and non ESBL-producing *K. pneumoniae* in an intensive care unit were 9.6% (7/73) and 13.6% (3/22), respectively [26].

In this study risk of mortality among those who suffered from bloodstream infection caused by ESBL producing *E. coli* or *K. pneumoniae* was not significantly different. This result was similar to the finding in another study in which they reported the hazard ratio was 1.65 (95% CI 0.75–3.64) [14]. In addition, this result was also similar to the findings in 2 other studies [13, 15].

Overall risk of mortality among exposed group was not statistically different with those un-exposed group. However, in stratification analysis showed that the exposed group had higher mortality risk as compared to un-exposed group especially in adult and elderly patients, patients with sign of leukocytosis, and patients treated with carbapenem. Most probably these 3 modifier effect variables were closely related with more severe clinical condition of the patients. In addition, the relationship between ESBL enzyme and virulence factor needs to be considered. The relationship between these two factors is not yet known with certainty. There are four factors that are suspected to have an effect on the increasing antimicrobial resistance relationship with virulence, namely (1) bacterial species; (2) specific virulence and resistance mechanisms; (3) environmental or ecological niche; and (4) the patient (immune system) [27]. Other studies suggest there is no convincing evidence that infections caused by ESBL producing bacteria are associated with poor outcomes (deaths) compared to those caused by non ESBL-producing bacteria, except in cases where antimicrobials were not provided optimally [9, 28].

## Limitation

Mortality of patients suffer from bloodstream infection caused by *E. coli* or *K. pneumoniae* might be due to infection of ESBL producing bacteria and also severity of the disease. In this study, severity of bloodstream infection was controlled by Pitt bacteremia score less than 4 for inclusion criteria for inception cohort. Mortality might also be due to progression or severity of underlying disease and/or co-morbid. Unfortunately, we were not able to control severity of these underlying diseases and co-morbid.

## Data Availability

All data generated or analyzed during this study are included in this published article and additional tables.

## Abbreviations

*E. coli*: *Escherichia coli*
*K. pneumoniae*: *Klebsiella pneuomiae*
ESBL: extended-spectrum β-lactamase
WHO: World Health Organization
HIV: human immunodeficiency virus
CI: convidence interval
LOS: length of stay
SIRS: systemic inflammatory response syndrome
SD: standard deviation
RR_strat_: relative risk from stratification analysis
RR_crude_: relative risk from total sample
RR_MH_: relative risk from Mantel-Haenzel analysis
HR: hazard ratio
